# Bactericidal Activity Does Not Predict Sterilizing Activity: The Case of Rifapentine in the Murine Model of *Mycobacterium ulcerans* Disease

**DOI:** 10.1371/journal.pntd.0002085

**Published:** 2013-02-28

**Authors:** Deepak V. Almeida, Paul J. Converse, Si-Yang Li, Sandeep Tyagi, Eric L. Nuermberger, Jacques H. Grosset

**Affiliations:** Center for Tuberculosis Research, Division of Infectious Diseases, Johns Hopkins University School of Medicine, Baltimore, Maryland, United States of America; Fondation Raoul Follereau, France

## Abstract

**Background:**

Since 2004, treatment of *Mycobacterium ulcerans* disease, or Buruli ulcer, has shifted from surgery to daily treatment with streptomycin (STR) + rifampin (RIF) for 8 weeks. For shortening treatment duration, we tested the potential of daily rifapentine (RPT), a long-acting rifamycin derivative, as a substitute for RIF.

**Methodology/Principal Findings:**

BALB/c mice were infected with *M. ulcerans* in the right hind footpad and treated either daily (7/7) with STR+RIF or five days/week (5/7) with STR+RIF or STR+RPT for 4 weeks, beginning 28 days after infection when CFU counts were 4.88±0.51.

The relative efficacy of the drug treatments was compared by footpad CFU counts during treatment and median time to footpad swelling after treatment cessation as measure of sterilizing activity. All drug treatments were bactericidal. After 1 week of treatment, the decline in CFU counts was significantly greater in treated mice but not different between the three treated groups. After 2 weeks of treatment, the decline in CFU was greater in mice treated with STR+RPT 5/7 than in mice treated with STR+RIF 7/7 and STR+RIF 5/7. After 3 and 4 weeks of treatment, CFU counts were nil in mice treated with STR+RPT and reduced by more than 3 and 4 logs in mice treated with STR+RIF 5/7 and STR+RIF 7/7, respectively. In sharp contrast to the bactericidal activity, the sterilizing activity was not different between all drug regimens although it was in proportion to the treatment duration.

**Conclusions/Significance:**

The better bactericidal activity of daily STR+RIF and especially of STR+RPT did not translate into better prevention of relapse, possibly because relapse-freecure after treatment of Buruli ulcer is more related to the reversal of mycolactone-induced local immunodeficiency by drug treatment rather than to the bactericidal potency of drugs.

## Author Summary

Until 2004, the treatment of Buruli ulcer (BU) was surgical excision followed by skin grafting. Now an 8-week daily regimen of streptomycin and rifampin (STR+RIF) is recommended by the World Health Organization, supplemented when necessary by surgical intervention. However, such an antimicrobial treatment is still a heavy burden in settings with limited public health infrastructure. Thus it would be beneficial to shorten the duration of treatment without reducing antibacterial activity. Among the more active drugs having the potential to reduce the duration of treatment is rifapentine (RPT), a rifamycin derivative with a much longer half-life than RIF, which permitted in mice the reduction of treatment duration for tuberculosis from 6 months to 3 months when it is substituted for RIF tuberculosis. We therefore compared in the mouse daily treatment of BU with STR+RIF and with STR+RPT. As expected, treatment with RPT was much more bactericidal than treatment with RIF, but surprisingly the relapse rate and the time to relapse after stopping treatment were not different between treatment groups. Such findings raise numerous issues on the mechanisms involved in the cure of Buruli ulcer and on the impact of bactericidal activities of rifamycin derivatives in this disease.

## Introduction

Buruli ulcer (BU) is a devastating skin infection caused by *Mycobacterium ulcerans* [1]. The standard antibiotic treatment combines rifampin (RIF) and streptomycin (STR) given for 8 weeks [2]. Since foci of the disease are primarily found in tropical areas of rural Africa and the majority of patients are children, a treatment with shorter duration would be more acceptable to the patients and facilitate the delivery of drugs in settings with limited public health infrastructure [3], [4]. In addition, shortening treatment duration will reduce the risks of oto- and nephro-toxicity and opportunities for bloodborne pathogen transmission associated with daily injection with streptomycin [5].

Replacing RIF with the long-lived rifamycin derivative rifapentine (RPT) in the standard first-line regimen for tuberculosis results in more rapid culture conversion (a measure of bactericidal activity) and a shorter treatment duration needed to prevent relapse (a measure of sterilizing activity). Consequently, the RPT-containing regimen allowed in mice a reduction of treatment duration from 6 months to 3 months [6], [7]. In the murine model of *M. ulcerans* disease, daily RPT at the lower dose of 5 mg/kg was shown to be as active as or even more active than daily RIF at 10 mg/kg [8]. In addition, RPT alone at 10 mg/kg was more active than RIF and STR given alone and almost as active as the STR+RIF combination [9]. Thus we hypothesized that the substitution of RPT for RIF would increase rifamycin exposure and antimicrobial activity, and consequently reduce the current duration of treatment for BU. To test this hypothesis the reduction of CFU counts during treatment and reactivation of the disease after cessation of treatment with STR+RPT given 5 days/week (5/7) were compared to those after the standard STR+RIF regimen in mice, given 5/7. Since in humans the standard treatment [10] is given 7 days/week (7/7) and STR+RIF regimen given daily 7/7 in mice was shown [11] more active than STR+RIF given 5/7, the 7/7 STR+RIF regimen was also included in the present experiment. Drugs were given for 4 weeks and doses were equivalent (similar AUC) to the human doses [12].

## Materials and Methods

### Antimicrobials

STR and RIF were purchased from Sigma (St. Louis, MO) and RPT was a gift from Sanofi-Aventis pharmaceuticals (Paris, France). Stock solutions of RIF and RPT were made in sterile 0.05% agarose solution and STR was prepared in sterile normal saline. All stock solutions were prepared weekly and stored at 4°C. RIF and RPT were administered, at 10 mg/kg body weight, orally using an esophageal cannula (gavage), and STR was administered by subcutaneous injections at 150 mg/kg body weight.

### Bacterial strain

Mice were infected with *M. ulcerans* 1615 (*Mu* 1615) (ATCC 35840), obtained from Dr. Pamela Small, University of Tennessee, that was originally isolated in Malaysia from a patient and is a part of the Trudeau collection [13], [14]

### Determination of minimum inhibitory concentration (MIC) for RIF and RPT

The *Mu* 1615 strain was grown on Middlebrook 7H11 + OADC (Becton-Dickinson, Sparks, MD) for 8–12 weeks. A uniform homogenous suspension was prepared from the colonies by suspending them in sterile phosphate buffered saline (PBS) with Tween 80 (0.05%) and vortexing them with sterile glass beads. The suspension was allowed to stand for 15–20 minutes till the large clumps settled, the supernatant was adjusted to an optical density at 600 nm (O.D. _600_) of 1, serially tenfold diluted from 0 to 10^−6^ in sterile PBS, and 0.5 ml of each dilution was inoculated on 7H11 agar + OADC plates containing twofold concentrations of RIF or RPT ranging from 0.007 to 2 µg/ml along with drug free controls. Plates were incubated at 32°C for 12 weeks.

### Footpad infection of mice

An aliquot of a twice-mouse-passaged *Mu 1615* strain stored at −80°C was thawed and inoculated in mouse footpads. Once the footpads were swollen to a lesion index of 2–3, defined as inflammatory footpad/hind foot swelling [15], mice were sacrificed and footpad tissue was harvested, minced and suspended in sterile PBS. The solution was vortexed briefly, allowed to stand for 30 minutes, and the supernatant was used for footpad infection. Prior to infection, the inoculum was checked qualitatively for acid-fast bacilli, serially diluted, and plated for CFU counts on selective 7H11 plates [9].

### Mouse model of treatment

The kinetic method developed by CC Shepard for assessing the activity of anti-leprosy drugs was used to assess drug activity [9], [16], [17]. In brief, 355 female BALB/c mice aged 4 to-6 weeks (Charles River, Wilmington, MA) were infected in the right hind footpad with 0.03 ml of the *Mu* 1615 suspension. After infection, mice were randomized to an untreated negative control group (n = 55) and two positive control groups treated with (i) STR+RIF given 5 days a week or 5/7 (n = 100) or (ii) STR+RIF given 7 days a week or 7/7 (n = 100). Finally, to assess the benefit of substituting RPT for RIF, the test group was the STR+RPT combination (n = 100), given five days a week or 5/7.

### Ethics statement

The study was conducted adhering to the Johns Hopkins University guidelines for animal husbandry and was approved by the Johns Hopkins Animal Care and Use Committee, protocol MO08M240. The Johns Hopkins program is in compliance with the Animal Welfare Act regulations and Public Health Service (PHS) Policy and also maintains accreditation of its program by the private Association for the Assessment and Accreditation of Laboratory Animal Care (AAALAC) International

### Assessment of treatment efficacy

Five mice from the untreated group were sacrificed the day after infection (D1) and 10 mice were sacrificed 26 days later (D26) at treatment initiation to establish baseline CFU counts in the footpads. The bactericidal activity of each drug regimen was monitored by performing weekly CFU counts from the foot pad of 5 mice per drug regimen and 5 untreated mice. In mice treated 5/7, the last gavage was on Friday morning and mice were sacrificed on the following Monday afternoon. In mice treated 7/7, the last gavage was on Sunday morning and mice were sacrificed the next day, on Monday afternoon. However to rule out any carryover by rifapentine (RPT), mice treated with streptomycin-rifapentine (STR+RPT 5/7) group were held for a wash-out period of one week after stopping treatment before being sacrificed.

The sterilizing potential of each drug regimen was monitored by determining the time to footpad swelling in negative control mice and in 20 mice treated for 1, 2, 3, and 4 weeks from each of the three treatment groups, according to the protocol given in Table 1. Footpad swelling was considered to have occurred when the footpad appeared inflamed to the naked eye, i.e., reached a lesion index of 2 [15]. Additionally to confirm whether the relapse was really due to multiplication of *M. ulcerans*, at least one mouse from each group which had appreciable swelling was cultured for CFU count.

**Table 1:** Scheme of the study.

Regimen	Number of mice sacrificed and of mice held for foot pad swelling after treatment completion, at the following time points						
	D -25	D0	Week 1	Week 2	Week 3	Week 4	Total
**Control**							
1) Untreated	5	10 (20)	5	5	5	5	35(20)
2) STR+RIF 5/7			5 (20)	5 (20)	5 (20)	5 (20)	20 (80)
3) STR+RIF 7/7			5 (20)	5 (20)	5 (20)	5 (20)	20 (80)
**Test**							
4) STR+RPT 5/7			5 (20)	5 (20)	5 (20)	5 (20)	20 (80)
**Total**	5	10 (20)	20(60)	20(60)	20(60)	20(60)	95(260)

The number in parenthesis indicates the number of mice kept for footpad swelling.

D-25 is the day after infection while D0 is the time of start of treatment.

Drugs and their doses were, STR = streptomycin, 150 mg/kg; RIF = rifampin, 10 mg/kg; RPT = rifapentine, 10 mg/kg.

Treatment was either 5 days a week (5/7) or 7 days a week (7/7).

For quantitative footpad CFU counts, mice were sacrificed by cervical dislocation and footpads were harvested after thorough disinfection with soap and sterile PBS followed by 70% alcohol swabs. Footpad tissue was then homogenized by fine mincing and suspended in 2 ml sterile PBS. Appropriate dilutions of 0.5 ml were plated on selective 7H11 plates in duplicate and incubated at 32°C for 12 weeks before CFU were enumerated.

#### End points

The activity of each treatment was assessed by comparing (i) the weekly reduction in the foot pad CFU counts of treatment groups with untreated controls and (ii) the median time to foot pad swelling after treatment cessation. The median time to footpad swelling was determined by checking the footpads of mice every week for 31 weeks after stopping treatment, i.e., 39 weeks after infection. If the median time to footpad swelling in treated mice exceeded that in untreated mice by no more than the duration of the treatment, i.e. 1, 2, 3, or 4 weeks, then the treatment was considered to be bacteriostatic. Longer median time to footpad swelling was indicative of bactericidal activity or prolonged post-antibiotic effect. Absence of swelling at the end of the follow-up period was indicative of sterilizing potential.

### Statistical Analysis

Survival analysis, with footpad swelling as the measurement, was performed using the Kaplan-Meier method [18]. The log rank test was used to determine the level of statistical significance when comparing survival curves of the different treatment groups with the control group. *p* values were two-tailed, and a value of *p*<0.05 was considered statistically significant. CFU counts were log-transformed before analysis. Culture-negative footpads were assigned a log value of 0. Group means for experimental treatment groups were compared with that of the standard treatment control by one-way analysis of variance with Dunnett's post-test. Paired t-tests were also used to compare groups of equal size. All analyses were performed with GraphPad Prism version 4.01 (GraphPad, San Diego, CA).

## Results

### MIC of RIF and RPT

For *M. ulcerans* strain 1615, the MIC of RIF was 0.25 µg/ml and the MIC of RPT was 0.125 µg/ml, one dilution lower than that of RIF.

### Reduction in the foot pad CFU counts

**Figure 1:**
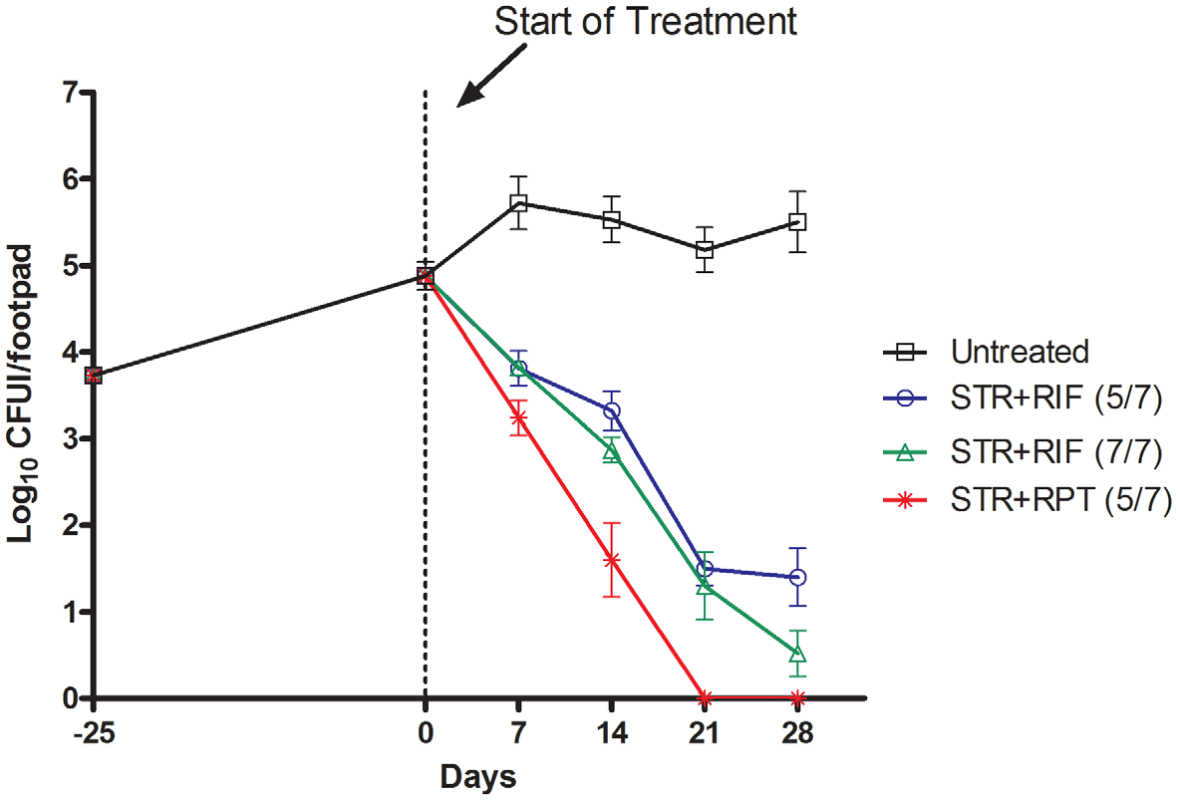
Decrease in log_10_ CFU counts in footpads. Decrease in the log_10_ CFU counts in the footpads of mice treated with, streptomycin+ rifampin for 5 days a week (STR+RIF 5/7), STR+RIF for 7 days a week (STR+RIF 7/7) and STR+ rifapentine (RPT) (STR+RPT 5/7) compared to untreated controls. The day after infection counts are shown at −25 (Mice were infected at D-26) and treatment was started after 25 days (D0).

The CFU counts by D-27, the day after infection, and by D0, the day on treatment initiation, and after 1, 2, 3, and 4 weeks of treatment are given in table 2 and figure 1.

**Table 2:** Reduction of *M. ulcerans* CFU counts in mouse footpads during treatment.

Regimen	Mean log_10_ CFU per foot pad ± SD at the following time points					
	D - 25	D0	Week 1	Week 2	Week 3	Week 4
**Control**						
1)Untreated	3.73±0.11	4.88±0.51	5.72±0.68	5.53±0.60	5.18±0.58	5.50±0.79
2)STR+RIF 5/7			3.81±0.45	3.32±0.50	1.50±0.43	1.40±0.74
3)STR+RIF 7/7			3.82±0.19	2.87±0.33	1.30±0.87*	0.52±0.59**
**Test**						
4)STR+RPT 5/7			3.24±0.45	1.60±0.96*	0***	0***

* no CFU detected in 1 out of 5 mice.

** no CFU detected in 2 out of 5 mice.

*** no CFU detected in 5 out of 5 mice.

D-25 is the day after infection while D0 is the time of start of treatment.

Drugs and their doses were, STR = streptomycin, 150 mg/kg; RIF = rifampin, 10 mg/kg; RPT = rifapentine, 10 mg/kg.

Treatment was either 5 days a week (5/7) or 7 days a week (7/7).

In the negative control untreated mice, CFU counts were 4.88±0.51, one log_10_ higher on treatment initiation (D0) than on the day after infection (D-27), and further increased to 5.72±0.68, by another one log_10_ during the following week, indicating that the organisms were in a replicating state on treatment initiation. Thereafter they plateaued at the 5.5 log_10_ level while the foot pad swelling that began at week 5 after infection, was present in almost all mice by week 6 after infection and progressively worsened up to week 8. The foot pad swelling occurred very late in few untreated mice, possibly because the foot pad inoculation did result in the implantation of a limited number of *M. ulcerans* in these mice.

At week 1 of treatment, the log_10_ CFU counts, were significantly (p<0.0001) reduced, by at least one log_10_, in all three treated groups of mice by comparison with baseline counts at treatment initiation. However they were similar in mice treated with STR+RIF 5/7 or 7/7, respectively while they were slightly lower (p>0.05) by about 0.5 log_10_, in mice treated with STR+RPT 5/7.

At weeks 2, 3, and 4, the CFU counts continued to decrease at a similar rhythm in mice treated with STR+RIF either 5/7 or 7/7, with a trend in favor of 7/7 over 5/7, though the difference was not statistically significant (p>0.05). At the end of 4 weeks of treatment, 2 out of 5 mice treated with STR+RIF 7/7 were culture-negative, while all mice in the group treated 5/7 were culture-positive. In mice treated with STR+RPT, the regression in CFU counts was logarithmic, and resulted in complete culture conversion to negative of the foot pads by week 3. The bactericidal activity of STR+RPT was thus spectacular and much more pronounced than that of STR+RIF even given 7/7 (p<0.01).

### Time to footpad swelling

**Figure 2:**
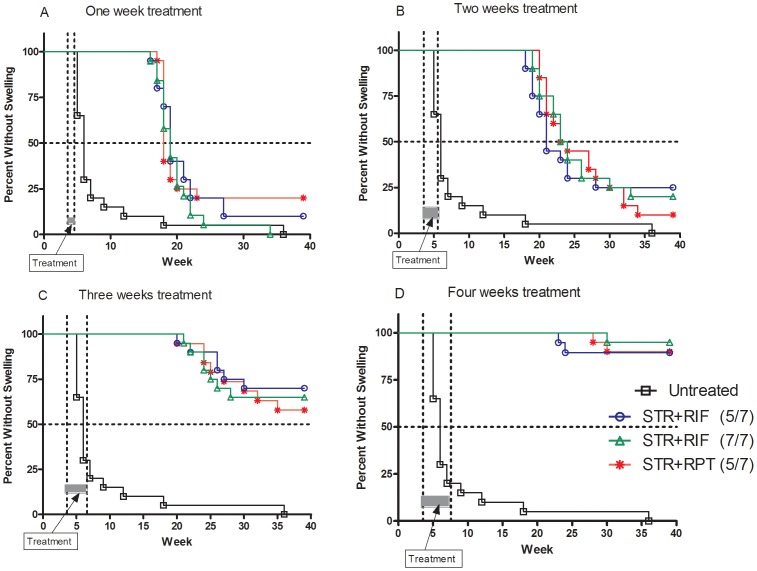
Time to footpad swelling. Post infection time to foot pad swelling in mice treated with either rifampin (RIF) or rifapentine (RPT) containing regimens for one week, two weeks, three weeks or four weeks. Untreated controls are shown in black squares, streptomycin (STR) + rifampin (RIF) for 5 days a week (5/7) in blue circles, STR+RIF for 7 days a week (7/7) in green triangles and STR+ rifapentine (RPT) (5/7) is in red asterisk.

The time to foot pad swelling after cessation of treatment with each of the different regimens given for 1, 2, 3, and 4 weeks is depicted in figure 2. This figure illustrates several important and rather unexpected findings. First, more than 50% (median) of untreated control mice exhibited foot pad swelling by week 6 after infection, providing the reference to which median time to swelling in treated mice were compared. Second, in all treated mice whatever the drug regimen and the duration of treatment, the median time to foot pad swelling was considerably delayed, by at least 12 weeks, compared to that in the untreated controls This finding reflects the potent bactericidal activity and/or the post-antibiotic effect of the tested drug regimens, even when they were administered for one week only. Third, as shown in figure 2, the longer the duration of treatment, the longer the time to swelling and the lower the proportion of mice that developed swelling during the 40 weeks of the experiment. The median times to swelling in mice treated for one and two weeks were 18 weeks and 20–22 weeks, respectively, indicating that a 2-week course of treatment was insufficient to prevent reactivation of the disease after treatment cessation. However, only 30 to 40% of mice treated for 3 weeks exhibited foot pad swelling at week 40, indicating that a treatment of 3 weeks duration was enough to cure more than half of the infected mice. Only a few mice treated for 4 weeks whatever the drug regimens exhibited foot pad swelling between weeks 25 to 30, suggesting that a treatment of 4 weeks duration is able to cure close to 100% of mice. Finally, and most surprisingly, none of the drug regimens tested over a period of 1 to 4 weeks was significantly better than the others in preventing foot pad swelling, e.g. reactivation of the disease. In other words, in the current experimental conditions, the RPT-containing regimen did not result in a higher rate of relapse-free cure than the RIF-containing regimens despite its much better bactericidal activity. It should also be noticed that the relapse-free cure rate was not better in mice receiving the STR+RIF regimen given 7/7 than in mice receiving the same regimen given 5/7.

## Discussion

The primary objective of the current study was to determine whether the increased rifamycin exposure associated with RPT administration would substantially increase the bactericidal activity and decrease the rate of reactivation after treatment cessation which is considered to be a measure of sterilizing activity, of the RIF-based regimen for the treatment of *M. ulcerans* infection in mice. As expected the STR+RPT combination resulted in better bactericidal activity than the STR+RIF combination. This was demonstrated by the weekly CFU counts and the culture conversion rate to negative by week 3 in all mice treated with the RPT-containing regimen whereas all mice treated with RIF-containing regimens were still culture-positive. Such a finding is in agreement with previous results [9]. However, contrary to our expectations [7], the more rapid culture conversion rate did not translate into a better cure rate in the RPT treated group as evidenced by the lack of significant differences with the RIF-treated groups in the time to foot pad swelling i.e., reactivation after treatment cessation. There was also no difference in time-to-swelling between mice treated with STR+RIF given either five or seven days a week. Such unexpected findings raise numerous issues related to the experimental model and the cure potency of the drug regimens used.

One possible explanation is technical and related to the one week delay after stopping treatment before cultures from the foot pad of the RPT-treated mice were performed. This procedure aimed to eliminate the risk of RPT carryover due to its long half-life of 15 hr. However, because of RPT's long half-life, *M. ulcerans* likely continued to be exposed to RPT during the days following treatment cessation. The better bactericidal activity of RPT after one, two and three weeks of treatment may thus result, at least in part, from continuation of RPT exposure after treatment cessation. If mice receiving RPT were effectively treated longer than mice receiving RIF, then that could explain why the bactericidal activity of the RPT-containing regimen was found better than that of RIF-containing regimens. But that cannot explain why the RPT-containing regimen was not better than the RIF-containing regimens in preventing relapse, except if there was no or limited correlation between bactericidal activity and sterilizing activity [19] against *M. ulcerans*. It has been suggested [20], [21] that the high protein binding of RPT, 97% versus 85% for RIF [7], may be partially responsible for its suboptimal activity. It is even possible that such a significant protein binding compensates for the slightly lower MIC of RPT (0.125 µg/ml) for *M. ulcerans* than that of RIF (0.25 µg/ml), especially if rifamycin activity is concentration-dependent [22]. Finally, it also is possible that the potential better sterilizing activity of rifapentine over rifampin which is well established in the murine model of tuberculosis [7] does not matter in the treatment of Buruli ulcer. For the cure of Buruli ulcer, the important thing may not be increasing the antimicrobial sterilizing potency but stopping the production of mycolactone to reverse as soon as possible the local mycolactone-induced immunodeficiency. In that role, rifampin and rifapentine, may have equivalent activity. Blocking the production of mycolactone for a sufficient length of time may, in fact, be more important than the speed of killing the microbe. Eventually, the cure after treatment likely results from the decline in mycolactone content and the development of a specific immune response, both phenomena being closely interlinked. One may even go further and consider that the concept of sterilizing activity which is crucial in the treatment of tuberculosis [23] and leprosy [24] is irrelevant in the treatment of Buruli ulcer, a toxin-derived disease like diphtheria and tetanus. All of these considerations give support to the WHO sponsored trial on the comparative curing effect on Buruli ulcer of the bactericidal STR + RIF combination and the less bactericidal RIF + clarithromycin combination [25]

Another point of concern is that the STR+RIF combination had only slightly more antimicrobial activity when it was administered on a daily basis (7 days a week) than when it was administered 5 days a week. This is in contradiction with the findings by Ji et al [11] that the relapse rate after 7 days a week treatment with the STR+RIF combination was significantly lower than after 5 days a week treatment with the STR+RIF combination. The likely explanation of such a difference between the Ji et al. results and ours may be found in the different microbial burdens on treatment initiation. In the Ji et al. experiment, the treatment of *M. ulcerans* infection was initiated when the infected mouse footpad was clearly swollen and the log_10_ CFU count has reached a peak at 6.09±0.19 per foot pad whereas in our experiment treatment was initiated when the foot pad was just beginning to swell and the log_10_ CFU count was only 4.88±0.51 per foot pad. The higher CFU count at treatment initiation might have biased the comparison in favor of a 7/7 regimen. Similar observations have been made in the murine model of tuberculosis in which the differences in the initial bacterial burden also influence the relapse rate after treatment cessation [26]. The differences between the Ji et al. data and our data also raise the issue of the more reliable experimental model to assess comparatively the bactericidal potential of drug regimens. Possibly the high microbial burden model with plateauing CFU and the low microbial burden model with CFU in the logarithmic period of growth provide insights that complement one another. As we do not know which model is the most predictive of relapse-free cure in humans , , better information would likely be gained by the simultaneous use of the two models. It is perhaps useful to remind ourselves of the limitations inherent to any experimental model in quoting Georges Canetti: “One should never forget the limitations of experimental studies in animals. They provide useful hypotheses and certain facts not observable in man, but in no case can they replace observations in man for the ultimate understanding of the disease (and its treatment) in human beings.” [27].
